# Supplementary material for: Understanding dietary behaviour change after a diagnosis of diabetes: A qualitative investigation of adults with type 2 diabetes
Source: PLoS One. 2022 Dec 12;17(12):e0278984. doi: 10.1371/journal.pone.0278984 (PMC9744287; doi:10.1371/journal.pone.0278984)
Supplement: S1 Table — (DOCX) [file pone.0278984.s001.docx]

**S1 Table**

**Journal:** PLOS ONE

**Manuscript title:** *Understanding dietary behaviour change after a diagnosis of diabetes: a qualitative investigation of adults with type 2 diabetes.*

Consolidated criteria for reporting qualitative studies (COREQ): a 32-item checklist for a qualitative study exploring the decision-making processes of dietary behaviour change after a diagnosis of type 2 diabetes (1).

|  | **No. Item** | **Guide questions/description** | **Reported on Page #** |
| --- | --- | --- | --- |
| **Domain 1: Research team and reﬂexivity** | | | |
| *Personal Characteristics* | | | |
| Interviewer/facilitator | 1 | Which author/s conducted the interview or focus group? | 8 |
| Credentials | 2 | What were the researcher’s credentials? E.g. PhD, MD | 8 |
| Occupation | 3 | What was their occupation at the time of the study? | 8 |
| Gender | 4 | Was the researcher male or female? | 8 |
| Experience and training | 5 | What experience or training did the researcher have? | 8 |
| *Relationship with participants* | | | |
| Relationship established | 6 | Was a relationship established prior to study commencement? | 7 |
| Participant knowledge of the interviewer | 7 | What did the participants know about the researcher? e.g. personal goals, reasons for doing the research | 7 |
| Interviewer characteristics | 8 | What characteristics were reported about the interviewer/facilitator? e.g. Bias, assumptions, reasons and interests in the research topic | 8 |
| **Domain 2: study design** | | | |
| *Theoretical framework* | | | |
| Methodological orientation and Theory | 9 | What methodological orientation was stated to underpin the study? e.g. grounded theory, discourse analysis, ethnography, phenomenology, content analysis | 6 |
| *Participant selection* | | | |
| Sampling | 10 | How were participants selected? e.g. purposive, convenience, consecutive, snowball | 7 |
| Method of approach | 11 | How were participants approached? e.g. face-to-face, telephone, mail, email | 7 |
| Sample size | 12 | How many participants were in the study? | 7 |
| Non-participation | 13 | How many people refused to participate or dropped out? Reasons? | 7 |
| *Setting* | | | |
| Setting of data collection | 14 | Where was the data collected? e.g. home, clinic, workplace | 6 |
| Presence of non-participants | 15 | Was anyone else present besides the participants and researchers? | 6 |
| Description of sample | 16 | What are the important characteristics of the sample? e.g. demographic data, date | 8 |
| *Data collection* | | | |
| Interview guide | 17 | Were questions, prompts, guides provided by the authors? Was it pilot tested? | 8 |
| Repeat interviews | 18 | Were repeat interviews carried out? If yes, how many? | 8 |
| Audio/visual recording | 19 | Did the research use audio or visual recording to collect the data? | 9 |
| Field notes | 20 | Were ﬁeld notes made during and/or after the interview or focus group? |  |
| Duration | 21 | What was the duration of the interviews or focus group? | 8 |
| Data saturation | 22 | Was data saturation discussed? | 10 |
| Transcripts returned | 23 | Were transcripts returned to participants for comment and/or correction? | 10 |
| **Domain 3: analysis and ﬁndings** | | | |
| *Data analysis* | | | |
| Number of data coders | 24 | How many data coders coded the data? | 10 |
| Description of the coding tree | 25 | Did authors provide a description of the coding tree? | No |
| Derivation of themes | 26 | Were themes identiﬁed in advance or derived from the data? | 9 |
| Software | 27 | What software, if applicable, was used to manage the data? | 9 |
| Participant checking | 28 | Did participants provide feedback on the ﬁndings? | 10 |
| *Reporting* | | | |
| Quotations presented | 29 | Were participant quotations presented to illustrate the themes/ﬁndings? Was each quotation identiﬁed? e.g. participant number | Yes  3. Results |
| Data and ﬁndings consistent | 30 | Was there consistency between the data presented and the ﬁndings? | Yes  3. Results |
| Clarity of major themes | 31 | Were major themes clearly presented in the ﬁndings? | 3. Results (major themes clearly presented under their own heading) |
| Clarity of minor themes | 32 | Is there a description of diverse cases or discussion of minor themes? | 3. Results (any minor themes formed within major themes and are discussed within the discussion of the major theme) |

**Additional information consistent with APA style reporting standards for qualitative research**

|  | **Reporting Recommendation** | **Reported on Page #** |
| --- | --- | --- |
| Method | Describe the researchers’ backgrounds in approaching the study, emphasizing their prior understandings of the phenomena under study | 8 |
|  | Describe any incentives or compensation | 9 |
| Analysis | Demonstrate consistency with regard to the analytic processes (e.g., analysts may use demonstrations of analyses to support consistency, describe their development of a stable perspective, interrater reliability, consensus) | 9-10 |
| Findings | Present synthesizing illustrations | Figure 1 |

**References**

1. Tong A, Sainsbury P, Craig J. Consolidated criteria for reporting qualitative research (COREQ): a 32-item checklist for interviews and focus groups. Int J Qual Health Care. 2007;19(6):349-57.
