# Supplementary material for: Understanding dietary behaviour change after a diagnosis of diabetes: A qualitative investigation of adults with type 2 diabetes
Source: PLoS One. 2022 Dec 12;17(12):e0278984. doi: 10.1371/journal.pone.0278984 (PMC9744287; doi:10.1371/journal.pone.0278984)
Supplement: S2 Table — (DOCX) [file pone.0278984.s002.docx]

**S2 Table**

**Journal:** PLOS ONE

**Manuscript title:** *Understanding dietary behaviour change after a diagnosis of diabetes: a qualitative investigation of adults with type 2 diabetes.*

Overarching topics that guided the development of the interview questions for a qualitative study exploring the decision-making processes of dietary behaviour change after a diagnosis of type 2 diabetes.

| **Interview guide topics** |
| --- |
| 1. The beliefs that guide individuals’ change in behaviour.   *Reorient participant*: Now let’s focus on your diet and foods choices after your T2D diagnosis. I’d like you now to tell me about your experiences after you were diagnosed with T2D.  Probe: What made you want to change your dietary behaviours? What was helpful? What wasn’t helpful? How did you feel? |
| 1. The strategies that dietitians use to facilitate dietary behaviour change.   *Reorient participant*: Let’s now focus now on your time with the dietitian, can you tell me about that and your experiences.  Probe: In what ways did your dietitian help you make dietary changes? In what ways did you help yourself make dietary changes? What was helpful? What wasn’t helpful? How did you feel? |
| 1. How individuals go about implementing such strategies into their lives.   Probe: How did you go about making those changes, the strategies or ‘things’ you did that helped you to action the changes? At any point, have you found yourself falling back to your old ways? What was helpful? What wasn’t helpful? How did you feel? |
| 1. Whether these strategies were helpful or not, and why.   Probe: What was helpful? What wasn’t helpful? How did you feel? |
| 1. What strategies individuals used to continue a change in behaviour and how they went about doing this   Probe: What changes do you still do since seeing a dietitian? How did the dietitian help you to make the decision to do the changes they suggested? |
