# Supplementary material for: Understanding dietary behaviour change after a diagnosis of diabetes: A qualitative investigation of adults with type 2 diabetes
Source: PLoS One. 2022 Dec 12;17(12):e0278984. doi: 10.1371/journal.pone.0278984 (PMC9744287; doi:10.1371/journal.pone.0278984)
Supplement: S3 Table — aBMI class classification according to WHO: [78]. bBMI class measurement taken at end of 3D-Study. cARIA = Accessibility/Remoteness Index of Australia. Outlines the participants remoteness in Australia based on postcode [79]. (DOCX) [file pone.0278984.s003.docx]

**S3 Table**

**Journal:** PLOS ONE

**Manuscript title:** *Understanding dietary behaviour change after a diagnosis of diabetes: a qualitative investigation of adults with type 2 diabetes.*

Characteristics and demographics of participants in a qualitative study exploring the decision-making processes of dietary behaviour change after a diagnosis of type 2 diabetes.

|  |  | n | (%) |
| --- | --- | --- | --- |
| Total participants | | 21 | (100) |
|  | Male | 9 | (43) |
|  | Female | 12 | (57) |
| Age | |  |  |
|  | Mean age (years) | 61.38 |  |
|  | Age range (years) | 36-75 |  |
| BMI^ab^ |  |  |  |
|  | Mean (kg/m^2^) | 30.01 |  |
| Dietetic consultations had after diagnosis to time of interview | |  |  |
|  | 1 | 3 | (14) |
|  | 2-4 | 11 | (52) |
|  | 5-7 | 6 | (29) |
|  | 13 | 1 | (5) |
| ARIA^c^ |  |  |  |
|  | Major city | 19 | (91) |
|  | Inner regional | 2 | (9) |

^a^BMI class classification according to WHO: [74].

^b^BMI class measurement taken at end of 3D-Study.

^c^ARIA = Accessibility/Remoteness Index of Australia. Outlines the participants remoteness in Australia based on postcode [75].
