# Supplementary material for: Understanding dietary behaviour change after a diagnosis of diabetes: A qualitative investigation of adults with type 2 diabetes
Source: PLoS One. 2022 Dec 12;17(12):e0278984. doi: 10.1371/journal.pone.0278984 (PMC9744287; doi:10.1371/journal.pone.0278984)
Supplement: S1 Fig — (DOCX) [file pone.0278984.s004.docx]

**S1 Fig**

**Journal:** PLOS ONE

**Manuscript title:** *Understanding dietary behaviour change after a diagnosis of diabetes: a qualitative investigation of adults with type 2 diabetes.*

Total 3D Study participant pool: n=225

9-month ‘Yes’ to being contacted in the future: n=158

12-month ‘Yes’ to being contacted in the future: n=5

Uncontactable or dropped out: n=56

‘No’ to being contacted in the future: n=6

Total willing to participate in further research: n=163

Had seen a dietitian after T2D diagnosis: n=98

Recruitment emails sent out: n=42

‘Yes’ Responses: n=28

Total number of participants for this study: n=21

Not willing to participate n= 1

Email not delivered: n=1

No response: n=12

Subsequent no response: n=5

Did not answer: n=2

Flow of participants from 3D-Study to current study for a qualitative study exploring the decision-making processes of dietary behaviour change after a diagnosis of type 2 diabetes.
