# Supplementary material for: Understanding dietary behaviour change after a diagnosis of diabetes: A qualitative investigation of adults with type 2 diabetes
Source: PLoS One. 2022 Dec 12;17(12):e0278984. doi: 10.1371/journal.pone.0278984 (PMC9744287; doi:10.1371/journal.pone.0278984)
Supplement: Attachment — Submitted filename: Revision plos one.docx [file pone.0278984.s006.docx]

The theme of the article is extremely relevant to health issues, especially at a time when the Covid-19 pandemic, for obvious reasons, occupied the concern of both researchers and the general population.

Understanding in depth how patients deal with changes in their lifestyle and diet can help to overcome the difficulties they experience.

Dietary re-education and an individual-centred approach constitute the main strategies of the Australian health system which, unfortunately, are still not enough. Understanding the reasons for this limitation is very important to improve approaches with patients.

The text presents a theoretical model about the motivation to change behavior and also points out its limitations, especially with regard to personal and unconscious aspects.

The qualitative approach and in-depth exploration of collected data is an appropriate way to develop new models and, consequently, more adequate ways to guide health professionals and their patients.

At the end of line 169, the final number of participating subjects can be indicated.

Line 202 - review dietitian spelling

Good improvement of the IBC model, expanding its scope.

Having only people over the age of 36 among participants may be a bias, younger adults may bring different data. In the conclusions I suggest highlighting the need for research with young adults.

I also believe that there should be stages for assimilating the diagnosis and different understandings and behaviors experienced by the same person, it seems to me a process with different stages that vary individually over time.

I also found it surprising not to mention the change in body image (weight loss), which would be an excellent motivation, especially depending on the person's affective life, if they are married, divorced, etc. Failure to mention economic difficulties to carry out a healthy diet also reveals the profile of the consumption pattern of the participants and of the country in which the study was carried out..

It is clear from the results that despite being experienced personally, the change in diet is a social process, whether in a proximal or virtual mode. Lonely people seem to be less motivated which reveals that all human experience is necessarily collective, even when this is not evident.

The person's mood did not emerge from the data but it probably has significant importance, I suggest pointing out in the closing remarks. Other emotional difficulties such as depression and anxiety must also play a part.

Environmental aspects are very relevant, both in their physical and relational aspects (obesogenic environment and facilitators/saboteurs).

The article is very relevant to knowledge in the field in developed and social welfare countries. Even so, it brings important insights about the subject and that can motivate research in other countries. This is important to relativize the universality of theoretical models that deal with individual differences, there are also differences between nations and even within them.

Clear and very informative text that can positively influence patients but, above all, healthcare workers. As the article is too long, I suggest that the authors, in a final review, select and remove or condense information and clarification that are repeated
