# Supplementary material for: Understanding dietary behaviour change after a diagnosis of diabetes: A qualitative investigation of adults with type 2 diabetes
Source: PLoS One. 2022 Dec 12;17(12):e0278984. doi: 10.1371/journal.pone.0278984 (PMC9744287; doi:10.1371/journal.pone.0278984)
Supplement: Attachment — Submitted filename: 1. Response to Reviewers_PONE-D-21-23420_final.docx [file pone.0278984.s007.docx]

Dear Dr Chenette,

Editor in Chief

PLOS ONE

**Re: Ms. No. PONE-D-21-23420.**

Thank you for the opportunity to revise and resubmit the manuscript, " Understanding dietary behaviour change after a diagnosis of diabetes: a qualitative investigation of adults with type 2 diabetes.", exclusively to PLOS ONE.

We thank you and the reviewers for your consideration and suggestions that we feel have improved the manuscript. We therefore submit the revised version for your consideration.

This document includes our responses to each piece of feedback. The new or edited text from the manuscript is inserted in italicized font below the relevant points to assist reviewers in evaluating changes made. The new page and line number are included in the table. All edits are in tracked changes. All authors have reviewed and approved the revised version of the manuscript.

We look forward to your response to this version.

Sincerely,

Dr Roshan Rigby

On behalf of the author team.

**Response to Reviewer Comments for PONE-D-21-23420**

| **New page and line #** | |
| --- | --- |
|  |  |
| JOURNAL REQUIREMENTS  1. Please ensure that your manuscript meets PLOS ONE's style requirements, including those for file naming. The PLOS ONE style templates can be found at  <https://journals.plos.org/plosone/s/file?id=wjVg/PLOSOne_formatting_sample_main_body.pdf> and  <https://journals.plos.org/plosone/s/file?id=ba62/PLOSOne_formatting_sample_title_authors_affiliations.pdf> | |
| **AUTHOR RESPONSE**  **Thank you for these templates.**   - **PLOS ONE style has been adhered to for formatting and file naming** | |
| JOURNAL REQUIREMENTS  2. Peer review at PLOS ONE is not double-blinded (https://journals.plos.org/plosone/s/editorial-and-peer-review-process). For this reason, authors should include in the revised manuscript all the information removed for blind review. | |
| **AUTHOR RESPONSE**   - **We have ensured there is no de-identified information on the revised manuscript.** - **The University name is now included in ethics statement within the manuscript text** | |
| *This study was approved by the Griffith University Human Research Ethics Committee (reference 2017/951).* | Page 7  Line 145-146 |
| JOURNAL REQUIREMENTS  3. We note that the grant information you provided in the ‘Funding Information’ and ‘Financial Disclosure’ sections do not match.  When you resubmit, please ensure that you provide the correct grant numbers for the awards you received for your study in the ‘Funding Information’ section. | |
| **AUTHOR RESPONSE**  **Thank you for identifying this. To clarify, this study did not receive specific funding. However, at the time of the study, the lead author was a PhD candidate who was supported by an Australian Government Research Training Program Scholarship.**   - **The ‘Funding Information’ section has been updated to include the Australian Government Research Training Program, awarded to Roshan R Rigby.** | |
| JOURNAL REQUIREMENTS  4. In your Data Availability statement, you have not specified where the minimal data set underlying the results described in your manuscript can be found. PLOS defines a study's minimal data set as the underlying data used to reach the conclusions drawn in the manuscript and any additional data required to replicate the reported study findings in their entirety. All PLOS journals require that the minimal data set be made fully available. For more information about our data policy, please see http://journals.plos.org/plosone/s/data-availability.  Upon re-submitting your revised manuscript, please upload your study’s minimal underlying data set as either Supporting Information files or to a stable, public repository and include the relevant URLs, DOIs, or accession numbers within your revised cover letter. For a list of acceptable repositories, please see http://journals.plos.org/plosone/s/data-availability#loc-recommended-repositories. Any potentially identifying patient information must be fully anonymized.  Important: If there are ethical or legal restrictions to sharing your data publicly, please explain these restrictions in detail. Please see our guidelines for more information on what we consider unacceptable restrictions to publicly sharing data: http://journals.plos.org/plosone/s/data-availability#loc-unacceptable-data-access-restrictions. Note that it is not acceptable for the authors to be the sole named individuals responsible for ensuring data access.  We will update your Data Availability statement to reflect the information you provide in your cover letter. | |
| **AUTHOR RESPONSE**  **Thank you for bringing this to our attention. We have now included the minimal data set that underlies the results and conclusion of our study. Data analysis was completed in a Microsoft Excel file and will be uploaded in the same format. The data has been deidentified in the file.**   - **Minimal data set underlying the results in the manuscript has been uploaded as Supporting Information.** | |
| JOURNAL REQUIREMENTS  5. Please review your reference list to ensure that it is complete and correct. If you have cited papers that have been retracted, please include the rationale for doing so in the manuscript text, or remove these references and replace them with relevant current references. Any changes to the reference list should be mentioned in the rebuttal letter that accompanies your revised manuscript. If you need to cite a retracted article, indicate the article’s retracted status in the References list and also include a citation and full reference for the retraction notice. | |
| **AUTHOR RESPONSE**  **We have reviewed all the references and have identified no changes.**  **Additional references added: [57] [58] [64] [76]**  57. Cockerham WC. Health lifestyle theory and the convergence of agency and structure. Journal of health and social behavior. 2005;46(1):51-67. doi: 10.1177/002214650504600105  58. Williams L. Research in dietetic practice and education: insights from the sociological perspective. 2016;73(3):217-9. doi: 10.1111/1747-0080.12301  64. Rigby RR, Mitchell L, Hamilton K, Ball L, Williams LT. Analyzing dietary behaviors self-reported by people with diabetes using a behavior change technique taxonomy. J Nutr Educ Behav. 2022;[Accepted for publication].  76. Wu YK, Berry DC. Impact of weight stigma on physiological and psychological health outcomes for overweight and obese adults: a systematic review. Journal of advanced nursing. 2018;74(5):1030-42. doi: 10.1111/jan.13511 | |
|  |  |
| REVIEWER #1 COMMENT  Reviewer #1: The theme of the article is extremely relevant to health issues, especially at a time when the Covid-19 pandemic, for obvious reasons, occupied the concern of both researchers and the general population.  Understanding in depth how patients deal with changes in their lifestyle and diet can help to overcome the difficulties they experience. Dietary re-education and an individual-centred approach constitute the main strategies of the Australian health system which, unfortunately, are still not enough. Understanding the reasons for this limitation is very important to improve approaches with patients. The text presents a theoretical model about the motivation to change behavior and also points out its limitations, especially with regard to personal and unconscious aspects. The qualitative approach and in-depth exploration of collected data is an appropriate way to develop new models and, consequently, more adequate ways to guide health professionals and their patients. | |
| **AUTHOR RESPONSE**  **We thank you for this comment.** | |
| REVIEWER #1 COMMENT  At the end of line 169, the final number of participating subjects can be indicated. | |
| **AUTHOR RESPONSE**  **The final number of participants has been added to the end of this section of the methods.** | |
| *The final number of participating subjects was 21.* | Page 8  Line 166-167 |
| REVIEWER #1 COMMENT  Line 202 - review dietitian spelling | |
| **AUTHOR RESPONSE**  **We have examined this line and cannot find the spelling error. The original line 202 stated: *“In what ways did your dietitian help you make dietary changes?”*. We have used spellcheck to ensure dietitian is spelt correctly throughout the manuscript text.** | |
| REVIEWER #1 COMMENT  Having only people over the age of 36 among participants may be a bias, younger adults may bring different data. In the conclusions I suggest highlighting the need for research with young adults. | |
| **AUTHOR RESPONSE**  **Thank you for addressing this aspect of our research as you have raised a valid point. We have added a sentence to the Strengths and Limitations section to suggest highlighting the need for research in young adults to address this gap.** | |
| *Our study did not capture the perspectives of young adults. Future research could explore this demographic as it may present different data compared to an older population.* | Page 41  Line 918-920 |
| REVIEWER #1 COMMENT  I also believe that there should be stages for assimilating the diagnosis and different understandings and behaviors experienced by the same person, it seems to me a process with different stages that vary individually over time. | |
| **AUTHOR RESPONSE**  **We agree that understanding a diagnosis is a process which changes over time. This process draws on concepts of coping mechanisms and acceptance of their condition. *How* this change occurs would be interesting to explore, such as how an individual experiences a diagnosis and how their understanding and beliefs change over time, including the factors that influence this process.**  **One theme, ‘post diagnosis realizations’ briefly touched on this, as T2D was a driver for their change, however this theme could be explored in further detail with more research to understand how this differs between people and how this process changes over time.**   - **Text added to Strengths and Limitations section** | |
| *Our data did not capture the processes of assimilating a diabetes diagnosis and how this might change over time for an individual, including how this understanding might impact on their dietary changes and diabetes health outcomes. Further research can explore the stages of understanding a diagnosis and the behaviours experienced by the same person to understand this process.* | Page 41  Line 903-907 |
| REVIEWER #1 COMMENT  I also found it surprising not to mention the change in body image (weight loss), which would be an excellent motivation, especially depending on the person's affective life, if they are married, divorced, etc. Failure to mention economic difficulties to carry out a healthy diet also reveals the profile of the consumption pattern of the participants and of the country in which the study was carried out. | |
| **AUTHOR RESPONSE**  **Thank you for this comment. Whilst we agree that body image might be a potential motivation for changing dietary behaviours, it did not come through strongly within this sample of participants. Some participants discussed wanting to lose weight to make themselves feel better which was reflected in the theme “to do it for themselves and others”, however this came through as a regaining of a sense of autonomy and control to manage their diabetes, rather than focusing on their body image. Exploring the influence of body image, weight loss and diabetes is an area that future research can investigate; we have included this suggestion as a future direction in the ‘Strengths and limitations’ section in the revised manuscript. That is an excellent point about the lack of mention of economic difficulty. Although not specifically stated, it did not appear that these participants had financial difficulty surrounding food and groceries. As the reviewer correctly states, this may reflect a more affluent profile of participants in this study and the developed country with a high-income economy in which the study took place. We have included this point as a limitation in the ‘Strengths and limitations’ section in the revised manuscript.**   - **Text added to ‘Strengths and limitations’ section to suggest future research exploring body image and diabetes.** | |
| *Weight stigmatisation and body image were not strongly identified in our participants. Given the known associations with adverse physiological and psychological health outcomes [new ref 76], future research can also explore the influence of body image, weight loss and diabetes.*   - **Text added to ‘Strengths and limitations’ section to acknowledge the limitation that the lack of mention of financial difficulty surrounding food and groceries may reflect the participants affluent profile.** | Page 41  Line 920-923 |
| *Future research can explore the lack of mention of financial difficulty amongst the participants, which may reflect a more affluent profile of participants as the study took place in a developed country with a high-income economy.* | Page 41  Line 901-903 |
| REVIEWER #1 COMMENT  It is clear from the results that despite being experienced personally, the change in diet is a social process, whether in a proximal or virtual mode. Lonely people seem to be less motivated which reveals that all human experience is necessarily collective, even when this is not evident. | |
| **AUTHOR RESPONSE**  **Yes, we agree with this statement. The change in diet experienced by our participants was a social process and had a variety of social influences, that although were experienced personally, had broader connections with others such as family members, friends, communities they were engaged with.** | |
| REVIEWER #1 COMMENT  The person's mood did not emerge from the data but it probably has significant importance, I suggest pointing out in the closing remarks. Other emotional difficulties such as depression and anxiety must also play a part. | |
| **AUTHOR RESPONSE**  **Yes, we agree that a person’s mood can have significant importance on their dietary intake and change. We have added a comment on this in the closing remarks in the revised manuscript.** | |
| *As the participants’ mood were not captured in this study, further research could explore the influence that mood and feelings associated with a type 2 diabetes diagnosis has on dietary intake and changes given the literature on emotion and decision making.* | Page 41  Line 915-918 |
| REVIEWER #1 COMMENT  Clear and very informative text that can positively influence patients but, above all, healthcare workers. As the article was too long, I suggest that the authors, in a final review, select and remove or condense information and clarification that are repeated | |
| **AUTHOR RESPONSE**  **We have undertaken a thorough review of the paper to reduce any redundancy and consolidate information where possible.** | |
|  |  |
| REVIEWER #2 COMMENT  I believe this manuscript deserves publication. It adds to the existing literature on the Integrated Behaviour Change (IBC) model, as the authors put it: “to incorporate key constructs that underpin multiple processes known to guide behavioural decision making”.  The authors make an excellent use of COREQ guidelines and principles, which I believe enhances the reporting of qualitative research methods and findings. This is not frequent and I would like to congratulate the authors on that choice. It clearly facilitates the reviewer process and provides reassurance on the methods.  I would also like to emphasize the merit of authors accounting for their paper’s limitations, and I agree with them, including assumptions such as homogeneity in dietician performance and service quality. | |
| **AUTHOR RESPONSE**  **We would like to express our gratitude for this comment of our research. Thank you.** | |
| REVIEWER #2 COMMENT  1. The authors claim (page 4, line 89) that “The exploration of such factors [individual difference factors and emotion determinants], alongside motivational, volitional, and implicit determinants of the IBC model, may pave the way for more elaborated integrated theories to be tested to advance knowledge on behavioural determinants and processes”. This is a valid claim. I agree with the authors that the IBC model is not enough in explaining behaviours (and thereafter lifestyles[**]). This has occupied the agenda of sociologists for some time now (which is not being acknowledged by authors in their text). See for example the work by Cockerham and other authors on Life Choices and Life Chances, which studied the importance of agency and structure in shaping lifestyles.  The authors do acknowledge to some extent the importance of structural variables (beyond agency). This becomes very evident on page 27, line 537 [“One participant who worked away at sea had limited control over the food provided on site, therefore his agency to choose food for his diabetes was restricted beyond his control”]. I would argue this offers the authors the opportunity to mention the importance of socially defined dimensions (structure) as constrains to free will and choice. The authors provide other examples of such factors, and although they group them up as “Individual difference factors” they clearly refer to social factors (work, social class, income level, physical environment) limiting the array of options available there for individual choice. For example, being able to pay for a dietician or for good quality food is clearly influencing outcomes here.  In brief, although the first paragraph in the methods section seems to account for social factors at the time of understanding how reality is really constructed, this somehow vanishes as you progress in the text.  [** When discussing dietary behaviours among T2D patients (which is a chronic condition patients will live with for the rest of their lives) we should be aware that such behaviours are precursors of lifestyles, and lifestyles are less easy to modify, largely constructed through repetition and reinforcement.] | |
| **AUTHOR RESPONSE**  **The focus of the study was to explore the individual’s influence of their dietary changes, and that of the dietitian, which is constructed through their reality. The behavioural science approach we took has a greater focus on agency compared to a sociological approach which includes the tensions, or interplay, between structure and agency. Had the interview guide included questions relating to broader social structures that influenced their dietary changes, this may have captured these elements more clearly, or would have emerged strongly from the transcripts. We have methodically revised the results and discussion and agree that there needs to be a stronger discussion on the social and structural constructs that may have influenced the dietary choices of our participants. We also must acknowledge this as a limitation to the IBC model that we used for our analysis, as the interplay of life choices (agency) and life chances (structure) are not strongly represented in the IBC model.** | |
| - **Paragraph added to discussion of implicit processes and obesogenic environments to discuss structure and agency** | |
| *From a sociological perspective, it is important to acknowledge the structural variables that influence individual choice and the interplay with agency. Although not explicitly discussed by the participants, there may be structural limitations affecting their dietary choices that are beyond their awareness or control. Such factors may include financial ability to seek out a dietitian and purchase certain food products, as well as accessibility to services or living conditions (new ref 57, 58). The interplay of agency and structure may be a limitation of the IBC model and future research may wish to consider these limitations when using the model from a behavioural science perspective.*  57. Cockerham WC. Health lifestyle theory and the convergence of agency and structure. Journal of health and social behavior. 2005;46(1):51-67. doi: 10.1177/002214650504600105  58. Williams L. Research in dietetic practice and education: insights from the sociological perspective. 2016;73(3):217-9. doi: 10.1111/1747-0080.12301 | Page 37  Line 797-804 |
| - **Sentence added to Strengths and Limitations section to capture the limitation the IBC model** | |
| *The interplay of agency and structure were not strongly represented in the IBC model which may limit the use of the model from a sociological perspective.* | Page 41  Line 913-915 |
| REVIEWER #2 COMMENT  2. This second issue is closely related to the first. In S3 Table “Characteristics and demographics of participants in a qualitative study exploring the decision-making processes of dietary behaviour change after a diagnosis of type 2 diabetes”, the authors account for GENDER, SELF-REPORTED SOCIAL CLASS, and INCOME levels. I do not really see the need for that when such variables are mostly absent in the analysis. Do not take me wrong. I feel very strong about the importance of such variables (see point 1) and I would have favoured a more detailed analysis of data according to gender and social class, for example. I am just pointing out that these are two key dimensions that somehow have been neglected by the authors as explanatory variables. | |
| **AUTHOR RESPONSE**  **Thank you for bringing this to our attention. Yes, we agree that these dimensions are important however have not been strongly embedded within our analysis and were included in the table to provide characteristics of the participants. As such, these elements have been removed from the S3 Table given their lack of influence on the analysis presented in this paper. Gender was discussed in our analysis and will remain the table.** | |
| Journal Note: While revising your submission, please upload your figure files to the Preflight Analysis and Conversion Engine (PACE) digital diagnostic tool, https://pacev2.apexcovantage.com/. PACE helps ensure that figures meet PLOS requirements. To use PACE, you must first register as a user. Registration is free. Then, login and navigate to the UPLOAD tab, where you will find detailed instructions on how to use the tool. If you encounter any issues or have any questions when using PACE, please email PLOS at figures@plos.org. Please note that Supporting Information files do not need this step. | |
| **AUTHOR RESPONSE**   - **Figure 1 and S1 Figure have been uploaded to PACE. No image problems were identified. A screenshot of the results of this process is provided below.**    | |
